# Percutaneous treatment of spontaneous left main coronary artery dissection using drug-eluting stent

**DOI:** 10.1186/1471-2261-14-191

**Published:** 2014-12-17

**Authors:** Christos Graidis, Dimokritos Dimitriadis, Vasileios Karasavvidis, Georgios Dimitriadis, Efstathia Argyropoulou, Fotios Economou, George Spiromitros, Antonios Antoniou, Georgios Karakostas

**Affiliations:** Department of Interventional Cardiology, Kyanous Stavros Hospital, Vizyis-Vyzantos 1 Street, Thessaloniki, 54636 Greece

**Keywords:** Spontaneous coronary artery dissection, Left main, Percutaneous coronary intervention

## Abstract

**Background:**

Spontaneous coronary artery dissection is a rare cause of ischemic heart disease and sudden death. Prompt diagnosis is of paramount importance, especially in cases when it manifests with ST elevation myocardial infarction (STEMI).

**Case presentation:**

We report a case of a 42 year-old woman, who presented with an anterior STEMI in a hospital without on-site percutaneous coronary intervention (PCI) facilities. She was transferred to our hospital and coronary angiography revealed a spontaneous dissection of the left main stem coronary artery (LM). The dissection was successfully managed with PCI.

**Conclusion:**

PCI appears to be a potential option, for the treatment of selected cases with spontaneous LM dissection, presenting with an acute coronary syndrome.

## Background

Spontaneous coronary artery dissection (SCAD) is a rare cause of ischemic heart disease and sudden death. The true incidence tends to be underestimated since most cases are diagnosed at autopsy, reflecting the significant mortality associated with this condition. The subtype of spontaneous LM coronary dissection is even less common (accounts for 6-12% of all primary coronary dissections), usually leading to sudden death or extensive infarction. Treatment options for SCAD include conservative treatment(medical), percutaneous coronary intervention(PCI) and coronary artery bypass graft surgery (CABG) still the optimal strategy for this disease process has not been clearly defined. We report here a case of spontaneous dissection of the LM coronary artery, with left anterior descending (LAD) and circumflex artery involvement (LCx), which occurred in a 42-year-old healthy woman and was unrelated with childbirth or other known risk factors. Rescue angioplasty with stenting was performed.

### Case Presentation

**Figure 1:**
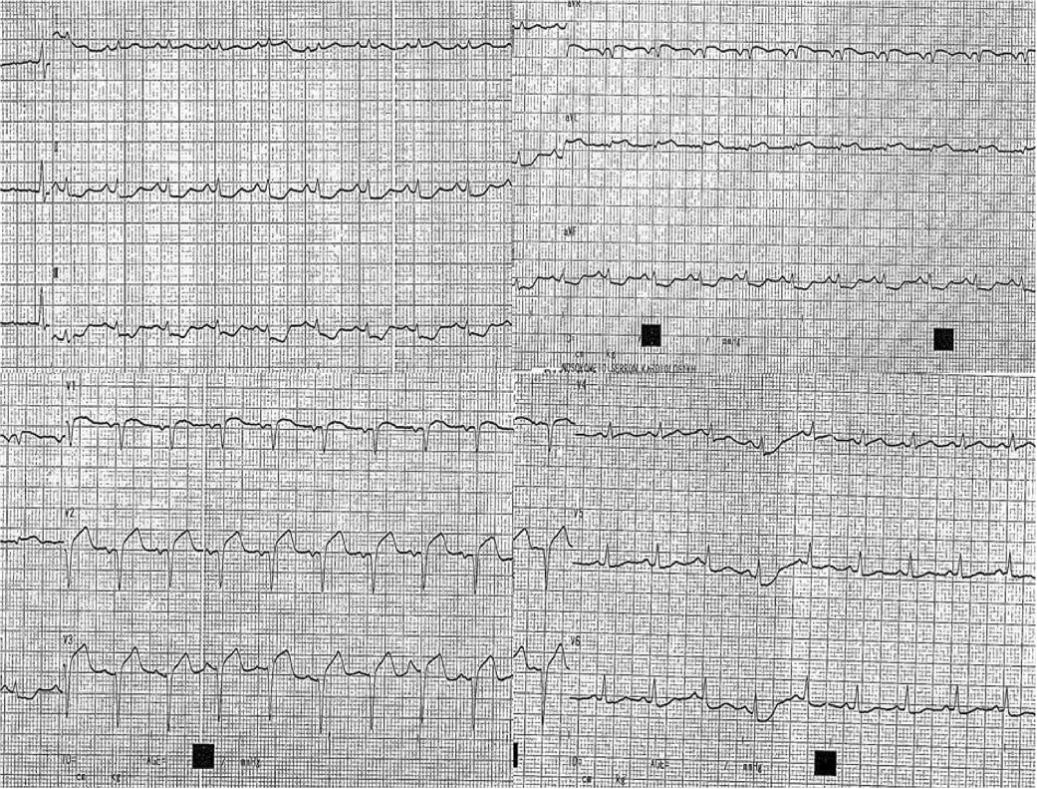
**ECG findings on admission were ST elevation in leads I, aVL, aVF, V2-V6.**

**Figure 2:**
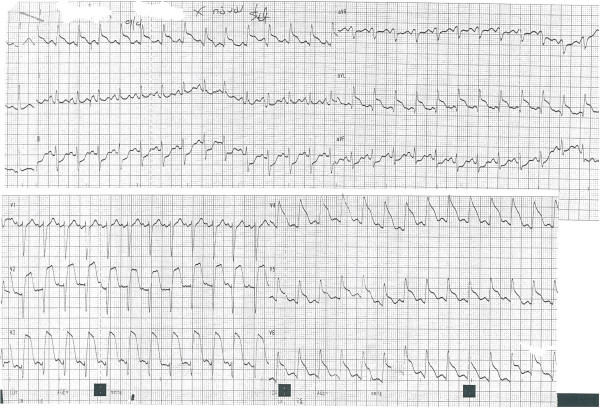
**On the eighth in-hospital day, the patient suffered another episode of substernal chest pain.** The ECG showed extensive ST elevation in leads I, aVL, V1-V6.

**Figure 3:**
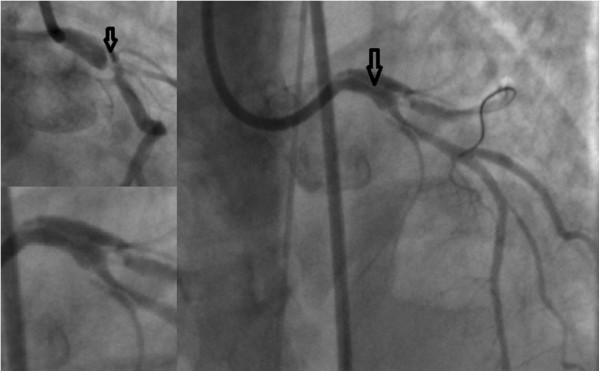
**Coronary angiography revealed the presence of a long dissection with intimal flap (arrows) of the left coronary trunk (LCT) with a double distal extension to the left anterior descending and circumflex arteries.**

**Figure 4:**
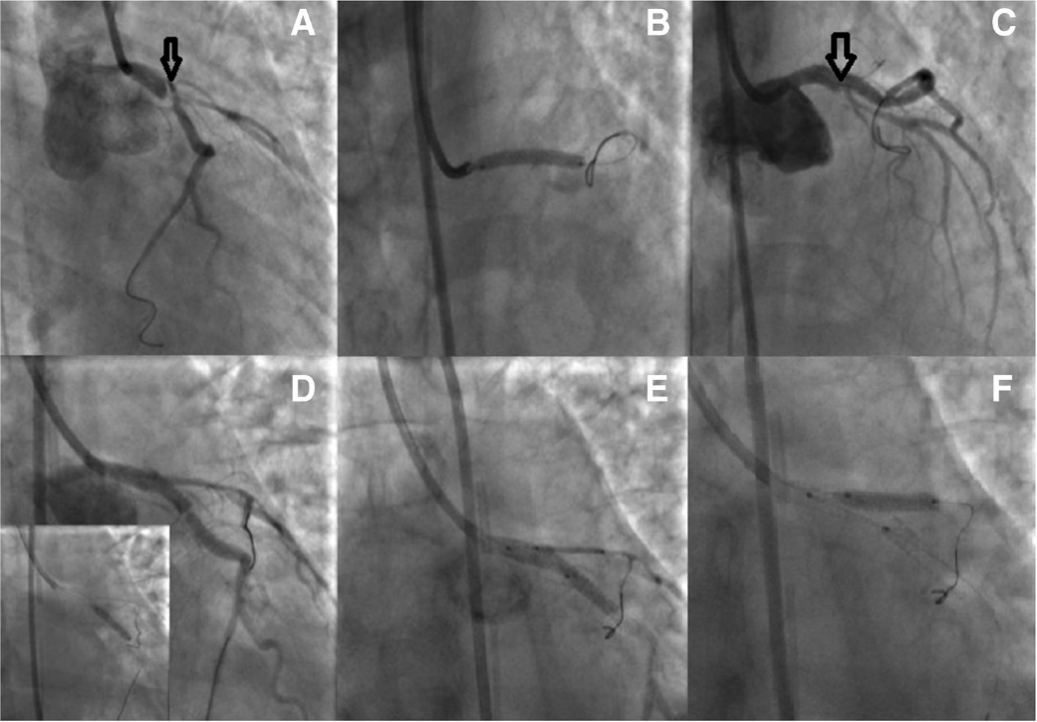
**PCI with TAP technique (T And Protrusion), step by step analysis. A**: The true lumen was crossed and the wire was placed in the distal Cx. **B**: A 3.0 × 24 mm Taxus Element stent was deployed at 14 atms into the left main and proximal LCx **C**: Due to the proximal sealing of the dissection (arrow), there was flow improvement in the LAD **D**: A second Taxus Element 3.0×16mm was deployed, due to residual stenosis distally to the implanted stent of the LCx, overlapping it **E**,**F**: The procedure was concluded by placing a Taxus Element 3.0 × 16 mm stent, into the LAD, with TAP technique (T And Protrusion).

**Figure 5:**
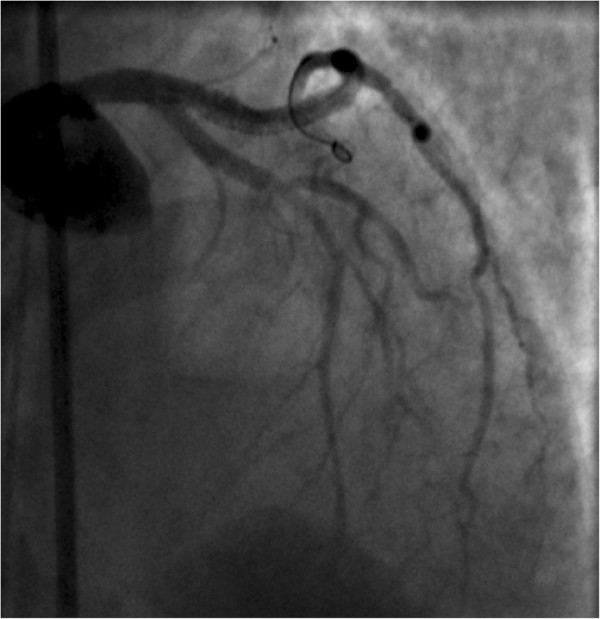
**Final result was excellent, with no residual dissection and TIMI 3 flow in to the LAD and LCx.**

**Figure 6:**
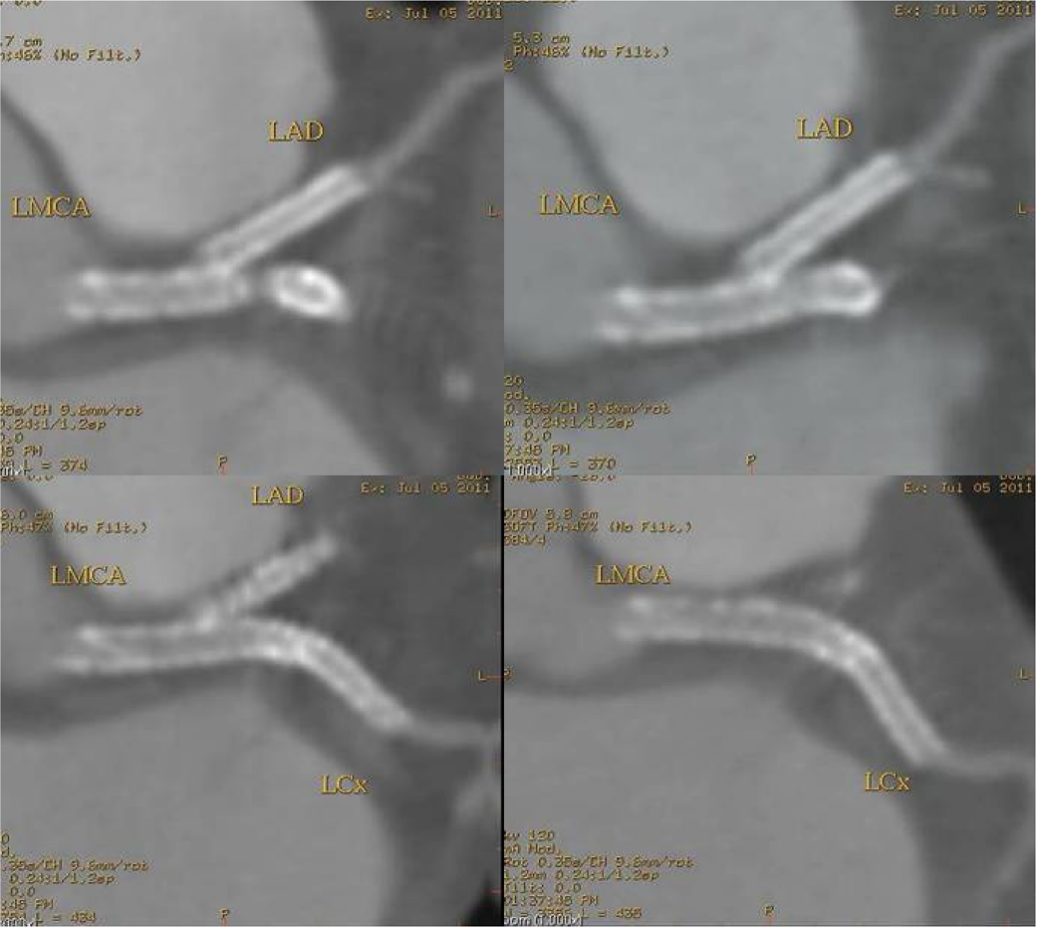
**A MSCT coronary angiography showed the absence of restenosis in the segments treated with stents at 3 months.**

A 42 years old Caucasian woman, without any previous medical history or any risk factors for coronary artery disease presented to a district general hospital, with no cardiac catheter laboratory facilities, complaining about a sudden-onset substernal chest pain lasting for the past 2 h. Her ECG findings on admission were ST elevation in leads I, aVL, aVF, V2-V6 (Figure 1). A thrombolytic agent was administered immediately, with regression of the angina and almost normalization of the ECG changes. The patient was not transferred to a hospital with PCI-capability for pharmaco-invasive strategy at that time, for unknown reasons. On the eighth in-hospital day, the patient complained of another episode of substernal chest pain, with hypotension and signs of left ventricular heart failure. The ECG showed extensive ST elevation in leads I, aVL, V1-V6 (Figure 2). Treatment with intravenous inotropic amines and re-thrombolysis (half-dose) was initiated and she was transferred to our hospital. On arrival to the coronary care unit, the patient was in cardiogenic shock. Echo on admission displayed anterior, apex and anteroseptum akinesia and severely impaired left ventricular function. The ejection fraction of the patient was 25% with moderate mitral regurgitation. She was electively intubated, an intra-aortic balloon pump (IABP) was advanced and she was urgently transferred to the cardiac catheterization laboratory. Coronary angiography revealed the presence of a long dissection of the left coronary trunk (LCT) with a double distal extension: a. Towards the anterior left descending coronary artery (LAD), the proximal third of which was occluded (TIMI-1 flow) and b. towards the circumflex artery (Cx) with slowed distal flow (TIMI-2 flow), (Figure 3). The right coronary artery (RCA) was normal. The diagnosis of a spontaneous coronary artery dissection was prompt, with these angiographic findings. The use of intravascular ultrasound (IVUS) or optical coherence tomography (OCT) was not plausible due to the emergency of the case. After a quick briefing with the heart team, immediate stenting was decided due to the hemodynamic compromise of the patient. Angioplasty and stenting with a drug eluting stent (DES) was therefore performed. After positioning a Q 3.5 SH 7Fr (Boston Scientific, Natick, MA, USA) guiding catheter using a Runthrough (Terumo Corporation, Tokyo, Japan) guide wire, the true lumen was crossed and the wire was placed in the distal Cx with free movement of the tip (Figure 4A). It was extremely difficult to place another guide wire to the distal LAD (despite multiple attempts and various J) and since there was a high risk of further extending the dissection, we decided to proceed with direct stenting of the left main. A 3.0 × 24 mm Taxus Element (Boston Scientific, Natick, MA, USA) stent was deployed at 14 atms into the left main and proximal Cx (Figure 5B). Due to the proximal sealing of the dissection, there was flow improvement in the LAD (Figure 4C). An Asahi Pro Water (Asahi Intecc, Japan) guide wire easily crossed the struts of the stent and was placed to the distal LAD, followed by inflations of two Sapphire (Orbus Neich Medical, B.V The Netherlands) 2.0 × 10 mm and a 2.5 × 15 mm balloons at the ostium and proximal of the LAD. A second Taxus Element 3.0X16mm was deployed, due to residual stenosis distally to the implanted stent of the Cx, overlapping it (Figure 4D). The procedure was concluded by placing a Taxus Element 3.0 × 16 mm stent, into the LAD, with TAP technique (T And Protrusion) (Figure 4E,F). The benefit of this combined technique is that the opportunity to first perform single-stenting is preserved, while side-branch stenting can be performed only if required. Final result was excellent, with no residual dissection and TIMI 3 flow in to the LAD and Cx. (Figure 5). She was discharged (day 8) on dual antiplatelet therapy, β-blocker, ACE inhibitor and statin. The patient remained asymptomatic at 3 months follow up and a MSCT coronary angiography showed the absence of restenosis in the segments treated with stents, followed by an improvement of left ventricular systolic function (Figure 6). A follow up cardiac catheterization angiography was recommended (class IIb) after 6 months according to European Society of Cardiology guidelines for high risk PCI (unprotected LM).

## Discussion

Spontaneous coronary artery dissection (SCAD) is an unusual but increasingly recognised cause of acute coronary syndromes and sudden cardiac death. This entity was first described in 1931 by Pretty during a post-mortem study of a 42-year old woman with sudden cardiac death [1]. The overall incidence of SCAD in angiographic series ranges from 0.07% to 1.1% [2–6]. It is likely to be underestimated, since in a significant number of published cases, the diagnosis was only made at autopsy. The mean age at presentation is 35 to 45 years [3, 6]. Seventy percent of the cases occur in women. One third of SCAD cases occur during the peripartum period, of which one third are in late pregnancy and two thirds in the early puerperal period. The LAD is the most common culprit vessel in women, whereas in men the RCA is more commonly affected [2–4]. Independently of gender, LAD is affected in 75% of the cases and RCA in 20% of the cases. Spontaneous dissection of the left main coronary artery (LMCA) accounts for 6-12% of all primary coronary dissections [3]. The circumflex artery was infrequently involved in men and women [7]. Multivessel involvement has also been described predominantly in women. The usage of modern techniques such as IVUS or OCT is of paramount importance in the diagnosis of SCAD. IVUS gained great importance in diagnosing SCAD especially of those that are angiographically unapparent. Typical IVUS features are the presence of intramural hematoma in the outer third of the media, visualization of the morphology of the vessel lumen, assessment of the intramural dissection and ensuring correct stent deployment. OCT being a new imaging modality provides extremely high resolution images of the coronary vessel. In cases of SCAD its exceptional spatial resolution provides a unique visualization of the intimal tear and the intramural hematoma. Although the pathophysiology remains unclear, it has been postulated that hemodynamic changes along with changes in the arterial wall architecture may predispose to intimal tears and intramural hematoma formation. The patients with SCAD have traditionally been divided into three groups: i) women in the peri-partum period or in oral contraceptive therapy, ii) patients with concomitant atherosclerotic coronary artery disease and iii) idiopathic. SCAD has also been associated with cocaine use, blunt trauma, Marfan’s syndrome, cystic medical necrosis, hypersensitivity vasculitis, coronary spasm, hypertension, fibromuscular dysplasia, after intense physical exercise and autoimmune thyroiditis [6].

There are no established guidelines available to guide treatment of patients with SCAD. Treatment options include medical therapy and revascularization with either CABG or PCI. The decision to pursue medical management or proceed to revascularisation with percutaneous intervention or coronary artery bypass grafting (CABG) should be individualized. Important factors to consider include location and extent of dissection, distal coronary blood flow, resolution or recurrence of symptoms following the initial event, hemodynamic stability and amount of myocardium at risk.

Conservative medical therapy is a reasonable approach in asymptomatic, stable patients with distal dissection or conserved coronary flow [7]. Medical therapy including aspirin, other antiplatelets, nitrates and beta-blockers has been successful in several cases with documentation of healing of the dissection on subsequent angiography. There are few data on the use of low molecular weight heparin or glycoprotein IIb/IIIa inhibitors [8]. Thrombolytic therapy is relatively contraindicated in SCAD due to the potential risk of worsening the dissection and contributing to expansion of the hematoma. Extension of dissection is possible [9], although successful use of thrombolysis has been described [10].

Revascularization for SCAD is warranted in those patients who present with ongoing ischemia refractory to medical treatment. Percutaneous coronary intervention [11] is the treatment of choice in patients with single-vessel involvement and signs of ongoing ischemia. Successful stenting for SCAD was first reported by Hong et al. [12] and since then percutaneous revascularisation has been increasingly performed resulting in very good long-term outcomes [13].

The interventionalist should be aware of the technical difficulties that may be encountered when dealing with such patients. It is essential to ensure that the guidewire is advancing in the true lumen. Passing the wire into the false lumen may occur more easily in these relatively non-fibrotic arteries were the use of OCT or IVUS would be a powerful weapon of the interventional cardiologist’s arsenal. Predilation with balloons should be avoided because it may lead to expansion of the dissection; instead direct stent implantation should be performed. A potential complication of stenting includes the extrusion of intramural thrombus up- or downstream of the stent, due to lack of fibrous tissue in these non-atherosclerotic vessels. That seems to have occurred in our case after implantation of the first stent with distal propagation of the dissection.

Surgical management is advisable in patients with left main stem or multiple vessel dissection jeopardising a large area of myocardium and when percutaneous intervention has failed [4, 14, 15]. Surgical revascularisation is also technically challenging, especially in identifying the true lumen, since grafting of the false lumen can have catastrophic consequences. Especially off-pump coronary artery bypass surgery minimises the risk of aortic dissection [15] and it is also preferable in pregnant women. Reported cases of left main spontaneous dissection are mainly treated with CABG [16].

To the best of our knowledge, there have only been a few reported cases of spontaneous left main dissection treated with percutaneous coronary stenting [16–19]. In our case, given the rapidly progressive nature of the dissection presenting as acute myocardial infarction, the delay from the transfer to our hospital, the unstable and critical hemodynamics at the time of procedure and the severity of LV dysfunction, it was felt that percutaneous stenting of the left main coronary artery to seal the entry point of the dissection would be the most expeditious therapy.

The prognosis of patients with SCAD has improved in recent years, likely due to increased diagnosis by cardiac catheterization and advances in treatment. In earlier series, mortality from spontaneous dissection was approximately 50%. With contemporary medical therapy, the rate of recurrent dissection is much lower and most patients are asymptomatic at follow up, with a 95% survival and 5% recurrent dissection rate [2–6].

## Conclusions

In summary PCI appears to be a potential option, for the treatment of selected cases with spontaneous left main coronary artery dissection, presenting with an acute coronary syndrome.

## Consent

Written informed consent was obtained from the patient for publication of this Case Report and any accompanying images. A copy of the written consent is available for review by the Editor-in-Chief of this journal.

## Abbreviations

STEMI: ST elevation myocardial infarction
SCAD: Spontaneous coronary artery dissection
PCI: Percutaneous coronary intervention
LCT: Left coronary trunk
LMCA: Left main coronary artery
LAD: Left anterior descending artery
RCA: Right coronary artery
LCx: Left circumflex artery
ECG: Electrocardiogram
CABG: Coronary artery bypass grafting.
